# Novel CaM-binding motif in its NudT9H domain contributes to temperature sensitivity of TRPM2

**DOI:** 10.1016/j.bbamcr.2018.12.010

**Published:** 2019-07

**Authors:** Ellen Gattkowski, Anke Johnsen, Andreas Bauche, Franziska Möckl, Frederike Kulow, Maria Garcia Alai, Trevor J. Rutherford, Ralf Fliegert, Henning Tidow

**Affiliations:** a The Hamburg Centre for Ultrafast Imaging & Department of Chemistry, Institute for Biochemistry and Molecular Biology, University of Hamburg, Martin-Luther-King-Platz 6, 20146 Hamburg, Germany; b Department of Biochemistry and Molecular Cell Biology, University Medical Centre Hamburg-Eppendorf, Martinistrasse 52, D-20246 Hamburg, Germany; c European Molecular Biology Laboratory Hamburg, Notkestrasse 85, D-22607 Hamburg, Germany; d MRC Laboratory of Molecular Biology, Francis Crick Avenue, Cambridge CB2 0QH, United Kingdom

**Keywords:** Calcium channel, Temperature sensor, Calmodulin-binding, TRPM2, 2′-deoxy-ADPR

## Abstract

TRPM2 is a non-selective, Ca^2+^-permeable cation channel, which plays a role in cell death but also contributes to diverse immune cell functions. In addition, TRPM2 contributes to the control of body temperature and is involved in perception of non-noxious heat and thermotaxis. TRPM2 is regulated by many factors including Ca^2+^, ADPR, 2′-deoxy-ADPR, Ca^2+^-CaM, and temperature. However, the molecular basis for the temperature sensitivity of TRPM2 as well as the interplay between the regulatory factors is still not understood.

Here we identify a novel CaM-binding site in the unique NudT9H domain of TRPM2. Using a multipronged biophysical approach we show that binding of Ca^2+^-CaM to this site occurs upon partial unfolding at temperatures >35 °C and prevents further thermal destabilization. In combination with patch-clamp measurements of full-length TRPM2 our results suggest a role of this CaM-binding site in the temperature sensitivity of TRPM2.

This article is part of a Special Issue entitled: ECS Meeting edited by Claus Heizmann, Joachim Krebs and Jacques Haiech

## Highlights

• Identification of a novel CaM-binding site in the NudT9H domain of TRPM2
• CaM acts as thermo-stabilizer for the isolated NudT9H domain
• CaM binding has an effect on the temperature-dependence of ADPR induced current and activation of TRPM2 by 2′-deoxy-ADPR
• Results suggest a role of this CaM-binding site in the temperature sensitivity of TRPM2.

## 1 Introduction

Transient receptor potential cation channel, subfamily M, member 2, also known as TRPM2, is a non-selective, Ca^2+^-permeable cation channel of the melastatin subfamily of TRP channels highly expressed in parts of the brain and phagocytic cells of the immune system [1,2]. While initial work on TRPM2 primarily addressed its role in cell death in response to oxidative and genotoxic stress [3,4], it becomes increasingly evident that the channel also contributes to immune cell functions like chemotaxis [5], chemokine [6] and cytokine secretion [2,7,8], regulation of oxidative burst [9] etc. In the brain TRPM2 is expressed not only in microglia and astrocytes but also in neurons [10,11]. In warm-sensitive neurons of the preoptic area of the hypothalamus TRPM2 contributes to the control of body temperature [12]. In the peripheral nervous system TRPM2 is expressed in a subset of somatosensory neurons and involved in perception of non-noxious heat and thermotaxis [13].

TRPM2 is regulated by a multitude of factors including intracellular nucleotides, calcium [[14], [15], [16]], temperature [17], phosphorylation [18] and pH [19]. A unique feature of TRPM2 is a C-terminal domain which is homologous to the mitochondrial ADPR pyrophosphatase NudT9 (NudT9H domain) [20]. Adenosine diphosphate ribose (ADPR), the substrate of NudT9 activates the channel, presumably by binding to this domain [20] whereas 2′-P-ADPR, a metabolite of the coenzyme NADP, acts as partial agonist [21]. 2′-deoxy-ADPR is an endogenous nucleotide that can be synthesized from nicotinamide mononucleotide (NMN) and deoxy-ATP via 2′-deoxy-NAD by the consecutive action of a NMN adenylyl transferase and the glycohydrolase CD38. It was recently found to act as TRPM2 superagonist that elicits much higher TRPM2 currents than ADPR at basal Ca^2+^ concentrations [22]. The activation of TRPM2 by ADPR is largely facilitated by intracellular Ca^2+^ or Ca^2+^ entering via the channel [14,15] whereas activation by 2′-deoxy-ADPR appears to be less sensitive to Ca^2+^ [22]. Removal of both intra- and extracellular Ca^2+^ completely prevents activation of the channel by ADPR [15]. Once activated, TRPM2 also shows Ca^2+^-dependent inactivation [15]. How the effects of Ca^2+^ on TRPM2 are mediated is currently not fully understood. Whole cell patch clamp experiments [15] and biochemical evidence [23] indicate a role of Ca^2+^-CaM in the sensitization to ADPR. Tong et al. found that calmodulin (CaM) binds to an IQ-like motif in the N-terminus of TRPM2 [23]. Csanády and coworkers in contrast proposed that Ca^2+^ is binding to a crevice near the cytosolic opening of the channel based on their work using excised inside-out patches from *Xenopus* oocytes expressing human TRPM2 [16]. In their recent cryo-EM structure of TRPM2 from *Nematostella vectensis* they located a Ca^2+^ binding site in between helices S2 and S3 [24]. Additionally, a recent study identified two EF loop motifs in the N-terminus of TRPM2, one of which seems to contribute to Ca^2+^ sensitization and activation of the channel by supraphysiological Ca^2+^ concentrations [25]. Very recently, cryo-EM structures of zebrafish TRPM2 [26] and human TRPM2 [27] in apo and ADPR-activated states provided exciting insights into the overall domain architecture as well as conformational changes occurring during channel activation. In addition, these studies revealed surprising differences with respect to ADPR-binding sites (MHR1/2 in drTRPM2 vs. NudT9H in hsTRPM2).

Besides its modulation by nucleotides and Ca^2+^, TRPM2 is also affected by temperature making it one of so far nine thermosensitive TRP channels (thermoTRPs). While activation of TRPM2 by heat alone requires high temperatures (threshold >47 °C) and results in rather small currents [28], exposure to temperatures >35 °C significantly potentiates the current evoked by ADPR [17]. The observation that the current in response to ADPR is affected by both Ca^2+^ concentration and temperature indicates that the regulation of TRPM2 by nucleotides, Ca^2+^ and temperature is interrelated.

Here we describe a novel CaM binding site in the NudT9H domain of TRPM2 that binds Ca^2+^-CaM upon partial unfolding at temperatures >35 °C. Binding of Ca^2+^-CaM to the NudT9H domain then prevents further thermal destabilization. In full-length TRPM2 replacement of the hydrophobic anchor residues of this CaM binding motif by alanine abrogates the increase in ADPR-induced current when temperature is shifted from room temperature to 37 °C. This indicates a role of this CaM binding site for the regulation of TRPM2 by temperature.

## 2 Materials and methods

### 2.1 Materials

All chemicals were of analytical grade and obtained from Roth (Karlsruhe, Germany) or SigmaAldrich (St. Louis, MO, USA), unless otherwise stated. Peptides were purchased from GL Biochem (Shanghai, China). Peptide identity was confirmed by LC-MS.

### 2.2 Enzymatic synthesis and purification of 2′-deoxy-ADPR

10 mM deoxy-ATP and 10 mM β‑nicotinamide mononucleotide were incubated in potassium phosphate buffer pH 7.5 (5 mM DTT, 20 mM MgCl_2_, 0.5 mg/ml bovine serum albumin) with recombinant human NMNAT-2 (R&D Systems) and NADase from *Neurospora crassa* (Sigma Aldrich) at 37 °C for 1 h. Product inhibition of NMNAT-2 was avoided by immediate turnover of 2′-deoxy-NAD by the NADase. The enzymes were removed by passing the solution through a centrifugal filter device with 10 kD cutoff (Sartorius, Göttingen, Germany). 2′-deoxy-ADPR was purified from the reaction mixture by preperative RP-HPLC using a volatile buffer (50 mM ammonium acetate, 0.05% acetic acid and 3% methanol) and a methanol gradient from 3% to 50% methanol on a 250 mm × 10 mm Luna C8 5 μm column (Phenomenex) equipped with a 10 mm × 10 mm guard cartridge containing a C8 ODS filter element (Phenomenex). A fraction containing 2′-deoxy-ADPR was collected, diluted and freeze dried from a large volume to avoid shifts in pH. After reconstitution in water the purified 2′-deoxy-ADPR was analyzed by analytical HPLC as described previously [22]. The peak area from the absorption at 260 nm was used to quantify the amount of 2′-deoxy-ADPR against a standard of ADPR (Sigma Aldrich).

### 2.3 Expression and purification of TRPM2 NudT9H domain

The TRPM2 NudT9H domain (residues 1236-1503) was expressed in *E*. *coli* strain Rosetta 2 (DE3) using a pET42a(+)-TEV vector. The protein was expressed with an N-terminal GST followed by His_6_-tag and a TEV protease cleavage site. The GST fusion was used to increase solubility of the protein. Bacteria were grown in Terrific Broth (TB) media containing 30 μg/ml kanamycin at 37 °C until a cell density of OD_600_ = 0.8 was reached. Protein expression was induced with 1 mM IPTG. Following protein expression overnight at room temperature, cells were harvested by centrifugation at 5000 ×*g* for 10 min. The supernatant was removed and the pellet frozen at −20 °C until further use.

For purification, cells were lysed by sonication, cleared from cell debris and the GST-His_6_-TEV-NudT9H fusion protein was purified by immobilized metal affinity chromatography (IMAC). After TEV protease cleavage, the fusion tags and the protease were removed using a second IMAC run. Finally, the NudT9H domain was further purified by size exclusion chromatography (SEC) using a buffer containing 25 mM Tris (pH 7.4), 150 mM NaCl, 10% (v/v) glycerol. Protein identity was confirmed by mass spectrometry.

### 2.4 Isothermal titration calorimetry (ITC)

The thermodynamic parameters were determined using a MicroCal ITC-200 isothermal titration calorimeter (Malvern Panalytcal, Malvern, UK) at 20–35 °C in a 25 mM Tris (pH 7.4), 150 mM NaCl, and 10 mM EGTA (Ca^2+^ free) or 2 mM CaCl_2_. For binding of CaM to NudT9H peptides, 335 μM peptide solution was titrated into the sample cell containing 20 μM CaM. For binding of CaM to intact NudT9H, 600 μM CaM was titrated into the sample cell containing 24 μM NudT9H. All samples were dialyzed extensively against the ITC buffer that was also used to dissolve the peptides, and centrifuged before use. Injection steps were 2 μL (first injection, 0.4 μL) with 150 s spacing and a stirring speed of 750 rpm. Baseline corrections were performed by titrating protein into sample buffer. Further data evaluation was done using the MicroCal Origin™ program. All titrations were run as triplicates and uncertainties in K_d_ are reported as fitting errors.

### 2.5 Circular dichroism (CD) spectroscopy

Far-UV circular dichroism (CD) measurements were made using a Jasco J-815 spectropolarimeter (Easton, MD, USA). Spectra were recorded from 260 nm to 195 nm (far-UV) using a 1 mm path length cell (Quartz-Suprasil, Hellma Analytics, Müllheim, Germany) and 4 μM protein. Ten scans were acquired using a scan rate of 20 nm/min. Buffer conditions were 25 mM sodium phosphate, 10 mM MgCl_2_, 15 mM NaCl, pH 7.2.

### 2.6 Differential scanning fluorimetry (nDSF)

The stability and thermal unfolding behavior of purified NudT9H in the presence and absence of ligands was followed using a nanoDSF differential scanning fluorimeter (Prometheus, NanoTemper Technologies, Munich) as previously described [29]. The intrinsic fluorescence at 330 and 350 nm after excitation at 280 nm is used to monitor the fluorescence change upon heat unfolding. Typically, 10 μl of a solution of the isolated NudT9H domain (0.2 mg/ml) and 1.0 mg/ml Ca^2+^-CaM were loaded in a capillary, and the unfolding was then measured at a heating rate of 1 °C/min. Buffer conditions were 25 mM Tris (pH 7.4), 150 mM NaCl, 2 mM CaCl_2_, 10% (v/v) glycerol. The first derivative of the unfolding curves was used to determine the transition midpoint. Simultaneously, protein aggregation was followed by light scattering.

### 2.7 Dynamic light scattering (DLS)

Measurements were performed using a DynaPro Nanostar (Wyatt Technology Corporation) instrument. Data were processed using Dynamics v.7 software. Protein concentrations were 20 μM NudT9H (+68 μM CaM) in a buffer containing 25 mM Tris (pH 7.2), 150 mM NaCl, 2 mM CaCl_2_, 3 mM MgCl_2_, 5% (v/v) glycerol. Samples were filtered through 0.22 μm centrifugal filters (Millipore) before the measurements. The acquisition time was 3 s with a total of 20 acquisitions and the heat rate was 1 °C/min.

### 2.8 Nuclear magnetic resonance (NMR) spectroscopy

All NMR spectra were acquired on a Avance III spectrometer operating at 600 MHz ^1^H frequency and equipped with cryogenic inverse probe and single-axis gradients. For binding studies, BEST-TROSY spectra [30] were acquired in a 25 mM Tris (pH 7.2), 150 mM NaCl, 5 mM CaCl_2_, 5% (v/v) glycerol at 298 K with concentrations of VL24 (NudT9H CaM-binding peptide) and uniformly labeled ^15^N-CaM of 250 μM and 130 μM, respectively. Spectra were acquired with 2048 and 256 data points in t2 and t1, respectively, covering spectral widths of 14.0 and 31.0 ppm. Spectra were processed using TopSpin 3.2 (Bruker) and figures were generated using NMR View [31].

### 2.9 Fluorescence spectroscopy

Fluorescence spectra of NudT9H peptide (VL24), CaM and the complex thereof were acquired using a Cary Eclipse Fluorescence Spectrophotometer (Agilent, Santa Clara, USA). Intrinsic Trp fluorescence of the peptide was excited at 280 nm and fluorescence emission spectra were measured from 310 nm to 400 nm. The peptide contains one Trp as potential anchor residue, while CaM does not contain any Trp residues. Protein concentrations were 2 μM VL24 and 4 μM CaM in a buffer containing 25 mM Tris (pH 7.2), 150 mM NaCl, with or without 2 mM CaCl_2_.

### 2.10 Cell culture

HEK293 cells were kept at 37 °C and 5% CO_2_ in DMEM with 4.5 g/l glucose and GlutaMax-I (Gibco) supplemented with 10% (v/v) fetal bovine serum (Biochrom). Cells were split 1:10 to 1:20 twice a week.

### 2.11 Site directed mutagenesis

The mutations W1355A and I1368A were inserted into the eukaryotic expression vector for TRPM2 (pIRES2-EGFP-TRPM2) [32] by two sequential rounds of QuikChange mutagenesis using the following primer pairs: for W1355A: 5′-CCCCATGGTCACGCGGGCGAGGCGGAACGAGGATGGAGCC-3′ and 5′-GGCTCCATCCTCGTTCCGCCTCGCCCGCGTGACCATGGGG-3′, for I1368A: 5′- GGAGCCATCTGCAGGAAGAGCGCAAAGAAGATGCTGGAAGTGCTG-3′ and 5′-CAGCACTTCCAGCATCTTCTTTGCGCTCTTCCTGCAGATGGCTCC-3′. The full open reading frame was confirmed by DNA sequencing (Eurofins MWG Operon, Ebersberg, Germany).

### 2.12 Whole cell patch-clamp experiments

For patch clamp experiments wild type HEK293 cells were transfected with expression vectors for either wild type TRPM2 or TRPM2 W1355A/I1368A 24 h before the experiments. To generate the transfection complex 2.5 μg of TRPM2 expression vector and 5 μl jetPEI reagent (PolyPlus Transfection, Strasbourg, France) were incubated for 30 min in a total volume of 250 μl (150 mM NaCl). Cells were detached from the culture bottle and adjusted to a density of 2.5 × 10^5^ cells/ml. After addition of the transfection complex cells were seeded at low density to 35 mm cell culture dishes. Directly before the experiment the culture medium was replaced by bath solution (in mM: 140 *N*‑methyl‑d‑glucamine (NMDG), 5 KCl, 3.3 MgCl_2_, 1 CaCl_2_, 5 d‑glucose, 10 HEPES, adjusted to pH 7.4 with HCl). The replacement of Na^+^ in the bath solution by NMDG (for which TRPM2 is impermeable) prevents cell rupture due to excessive current and the characteristic IV curve helps to distinguish activation of the nonselective cation channel from cells becoming leaky. Pipettes, pulled from thin-walled borosilicate glass capillaries (1.10 mm × 1.50 mm × 80 mm) using a Sutter P-97 horizontal puller, had a resistance between 1.5 MΩ and 5 MΩ and were filled with intracellular solution (in mM: 120 KCl, 8 NaCl, 1 MgCl_2_, 10 EGTA, 5.6 CaCl_2_, 10 HEPES, adjusted to pH 7.2 with KOH). The intracellular solution contains 200 nM free Ca^2+^ (calculated using MaxChelator, http://maxchelator.stanford.edu/CaEGTA-NIST.htm). Whole cell currents were recorded using a HEKA EPC-10 amplifier and PatchMaster software (HEKA Elektronik, Lamprecht, Germany). To record TRPM2 activity, repetitive voltage ramps from −85 mV to +20 mV over 140 ms were applied every 5 s for 450 s. Before and in between ramps cells were clamped at −50 mV. Series resistance compensation was set to 70%. For temperature control the 35 mm dishes were placed inside an open culture dish incubator (DH-35i, Warner Instruments) connected to a single channel heater controller (TC-324C, Warner Instruments). Cells were continuously perfused at a flow rate of 0.3 ml/min with bath solution pre-warmed in a water bath.

### 2.13 Biotinylation of TRPM2 on the plasma membrane

HEK293 cells were transfected with either pIRES2-EGFP-TRPM2, pIRES2-EGFP-TRPM2 W1355A I1368A or pIRES2-EGFP. 7.5 μg of the expression vector was mixed with 6 μg of Plus reagent (Invitrogen) in 1.5 ml Opti-MEM (Gibco) and incubated for 5 min at RT. After addition of 18 μl Lipofectamine LTX (Invitrogen) the complex was incubated for an additional 30 min at RT. Cells were grown in T25 cell culture bottles to ~80% confluency. At the day of transfection the medium from the cells was replaced by DMEM containing the transfection complex. After 5–6 h half of the medium was replaced by fresh medium. Cells were kept at 37 °C and 5% CO_2_ for an additional 43 h before they were washed with D-PBS (with Ca^2+^ and Mg^2+^) and incubated for 30 min at room temperature with 1 mg/ml EZ-Link Sulfo-NHS-LC-Biotin (Pierce/Thermo Scientific). After biotinylation cells were detached with 2 mM EDTA in D-PBS (without Ca^2+^ and Mg^2+^) and collected. Membrane proteins were isolated using the ProteoExtract Native Membrane Protein Extraction Kit (EMD Millipore) according to the manufacturer's instructions and protein concentration determined by Bradford assay. For pulldown of biotinylated proteins, 100 μl of the 50% slurry of NeutraVidin Agarose Beads (Pierce/Thermo Scientific) were added to 600 μg of the membrane protein and rotated overhead for 12 h. Afterwards both, the biotinylated proteins from the pulldown (from 300 μg of membrane protein) and total membrane proteins (10 μg), were incubated for 7 min at 75 °C in SDS loading buffer, separated on a 4–15% Protean precast SDS–PAGE gel (BioRad), and transferred onto a PVDF membrane. A prestained marker indicated where to cut the membrane to separately detect TRPM2 and Na^+^/K^+^-ATPase. The part of the membrane containing the high molecular weight range was probed using an anti-hsTRPM2 antibody from rabbit (Novus #nb500-241 at 1:50000), the other part of the membrane was probed with an anti-Na^+^/K^+^-ATPase antibody from rabbit (Cell Signaling Technology #3010 at 1:1000). Both primary antibodies were detected by an HRP-conjugated anti-rabbit secondary antibody (Dianova #111-035-045 at 1:10000). Chemiluminescence was recorded using a LAS-4000 Intelligent Dark Box (Fujifilm, Tokyo, Japan) after incubation of the membranes with a mixture of SuperSignal West Dura/Pico chemiluminescent substrates (Pierce/Thermo Scientific). Fiji (http://fiji.sc) was used for densitometric analysis of the images [33].

### 2.14 Statistical analysis

For statistical analysis GraphPad Prism (v7.04 and v8.0.1; GraphPad Software) was used. Currents obtained from whole cell recordings tend to be positively skewed and were therefore log-transformed to achieve normality. After transformation all groups were normally distributed as assessed by D'Agostino & Pearson normality test. Data were tested by one-way ANOVA. p values from post-hoc comparisons between groups were adjusted for multiple comparison using Bonferroni correction. A significance level of α = 0.05 was adopted.

## 3 Results and discussion

### 3.1 Identification of a new CaM-binding domain in the TRPM2 NudT9H domain

**Fig. 1:**
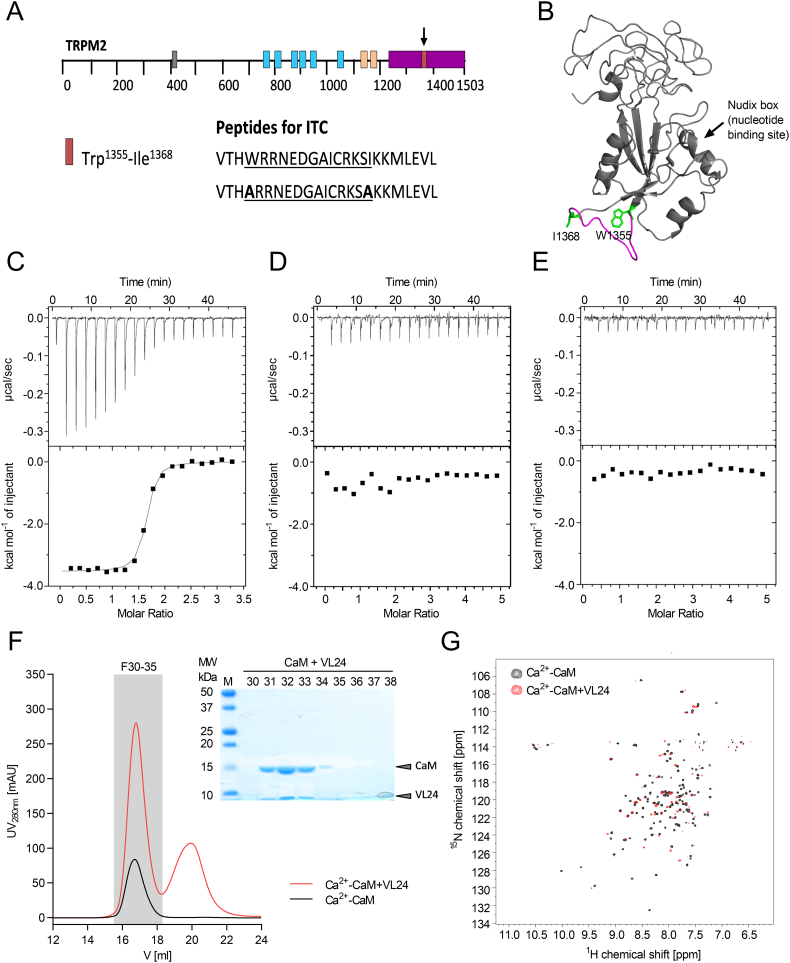
Identification of a new CaM-binding motif in the TRPM2 NudT9H domain. (A) Domain organization of the TRPM2 channel indicating the location of functional motifs in the amino acid sequence (grey box: known IQ-like motif, blue boxes: transmembrane helices, sand box: coiled coil domains, purple box: NudT9H domain, orange box: new CaM binding motif). The putative new CaM binding motif is marked by an arrow. The peptides used for ITC, NMR and fluorescence measurements are shown below. Peptide VTHWRRNEDGAICRKSIKKMLEVL is denoted VL24 throughout this study. (B) Structure of human NudT9H domain (from full-length hsTRPM2 cryo-EM structure (pdb:6MIX)). The putative CaM binding motif (magenta loop with anchor residues in green) is located on the surface of the NudT9H domain. The location of the Nudix box, the putative ADPR binding site is indicated by an arrow. (C–E) ITC measurements of CaM and the respective peptide. (C) Binding of CaM to peptide VL24 in the presence of 2 mM Ca^2+^ revealed a K_d_ of 110 nM (±18 nM). (D) VL24 with mutated anchor residues does not bind to Ca^2+^-CaM. (E) VL24 does not bind to CaM in the absence of Ca^2+^. (F) Purification of the complex of Ca^2+^-CaM and VL24 by SEC. Chromatography profiles of CaM alone and in complex with the peptide (left) and the SDS-PAGE of the corresponding complex fractions (right). (G) BEST-TROSY NMR spectrum of ^15^N-Ca^2+^-CaM alone (black) and in complex with access unlabelled VL24 peptide (red). Large perturbation of chemical shifts or disappearance of many peaks in the complex indicates significant conformational changes upon binding.

As the molecular mechanism for the regulation of TRPM2 by CaM is still poorly understood, we set out to discover/investigate potential CaM-binding sites on TRPM2. Although a single consensus sequence for CaM-binding site does not exist and bioinformatics detection of CaM-binding sites is complicated [34], most CaM-binding sequences contain a characteristic pattern of two hydrophobic anchor residues separated by 10, 14 or 16 residues (e.g. 1-10/1-8-14 spacing) flanked by positively charged residues [35,36]. Using this information, we identified residues 1355–1368 in human TRPM2 as a potential CaM-binding site (Fig. 1A). This site is also partly predicted as putative CaM-binding motif by Calmodulin Target Protein Database search tool (http://calcium.uhnres.utoronto.ca/ctdb/ctdb/sequence.html) established by the Ikura group [37]. This site is located in the NudT9H domain and most likely forms a surface-exposed loop connecting two beta-strands (Fig. 1B).

Using isothermal titration calorimetry (ITC), we confirmed tight binding of Ca^2+^-CaM to this binding peptide (from now denoted VL24) with a K_d_ of 110 nM (±18 nM). Mutating the potential anchor residues to Ala (W1355A I1368A) abolished binding to Ca^2+^-CaM. In addition, no binding could be detected between apo-CaM (absence of Ca^2+^) and VL24 (Fig. 1C–E). These experiments confirm that NudT9H contains a previously unknown CaM-binding motif that has the potential to bind tightly to Ca^2+^-CaM using W1355 and I1368 as anchor residues with 1–14 spacing. We reconstituted the Ca^2+^-CaM + VL24 complex and purified it by size-exclusion chromatography (SEC). The fact that the Ca^2+^-CaM + VL24 complex elutes slightly later than CaM alone (Fig. 1F) suggests that the complex has a more compact structure compared to the rather elongated dumbbell-shaped structure of Ca^2+^-CaM [38]. Upon binding to target peptides, Ca^2+^-CaM often wraps around the alpha-helical target peptides with its two EF-hand domains [[39], [40], [41], [42]] (reviewed in [36]). NMR spectroscopy was used to investigate the structural changes on a residue-specific level. Upon binding to VL24 almost all resonances of ^15^N-Ca^2+^-CaM show significant changes (either chemical shift perturbation or loss of signal) (Fig. 1G). This indicates that both domains are involved in binding, consistent with a mechanism where Ca^2+^-CaM wraps around the NudT9H-derived VL24 peptide upon binding.

### 3.2 CaM acts as thermo-stabilizer for the isolated NudT9H domain

**Fig. 2:**
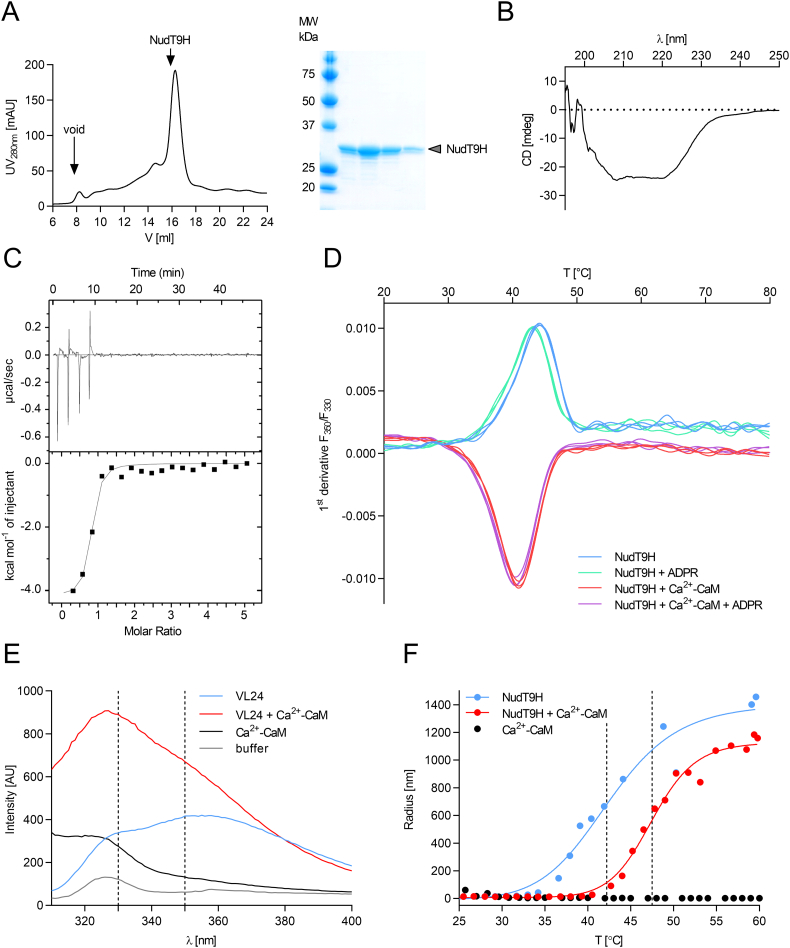
CaM acts as thermo-stabilizer for the isolated NudT9H domain. (A) Successful purification of the isolated NudT9H domain shown by SEC and the SDS-PAGE of the corresponding NudT9H-containing fractions. (B) CD spectrum of the isolated NudT9H domain indicates a predominantly α-helical fold. (C) Binding of Ca^2+^-CaM to the isolated NudT9H domain at 35 °C measured by ITC. (D) Thermal stability investigation of NudT9H by nDSF. Trp fluorescence of NudT9H suggests a temperature-dependent binding of CaM in the presence of Ca^2+^. Trp residues in NudT9H were exposed while unfolding at increasing temperatures independent of ADPR (blue and green curves) occurred. Ca^2+^-dependent binding of CaM to the unfolding NudT9H domain leads to the shielding of Trp residues (red curves) independent of ADPR (violet curves). (E) Trp fluorescence spectra of VL24 peptide alone and in complex with access Ca^2+^-CaM. The peptide contains one Trp as potential anchor residue, while Ca^2+^-CaM does not contain any Trp residues. The observed blue shift in the complex indicates that the Trp residue in the peptide is buried in a hydrophobic environment upon CaM-binding, indicating that this Trp residue in the NudT9H domain is responsible for the effect observed by nDSF (panel (D)). (F) Thermo-stabilizing effect of the NudT9H/Ca^2+^-CaM complex observed by dynamic light scattering (DLS). The isolated NudT9H domain alone (blue) aggregates at 42 °C while the complex (red) is stabilized to 47 °C. Ca^2+^-CaM alone (black) does not aggregate up to 60 °C.

Having identified a novel CaM-binding motif in the NudT9H domain (of TRPM2) we next set out to investigate the interaction between CaM and the intact NudT9H domain. We recombinantly expressed and purified NudT9H domain (comprising residues 1236–1503) (Fig. 2A). Its far-UV CD spectrum indicated a predominantly alpha-helical fold as expected from the homologous NudT9 [43] (Fig. 2B). When investigating the binding of Ca^2+^-CaM to NudT9H by ITC (as previously for the VL24 peptide), we could not detect binding at room temperature. At 35 °C, however, clear binding with a K_d_ of 470 nM could be detected (Fig. 2C). Based on these results and the homology model, we speculated that the CaM-binding motif might not be fully accessible for CaM-binding in natively folded NudT9H, but could become accessible upon (partial) unfolding of NudT9H at elevated temperatures.

To further investigate this hypothesis, we investigated the unfolding of NudT9H in various ligand-bound states by differential scanning fluorimetry (nDSF) (Fig. 2D). This method measures changes in intrinsic tryptophan fluorescence (F350/F330) upon thermal unfolding and usually reports on increased solvent exposure of previously buried Trp residues upon unfolding. The nDSF profiles of NudT9H in the presence and absence of ADPR are very similar with a T_m_ of about 42 °C, ruling out a stabilizing effect of ADPR on NudT9H. When performing these measurements in the presence of Ca^2+^-CaM, the nDSF profiles changed completely, now showing a decrease in the fluorescence emission at 350 nm (F350) and an increase in F330. This indicates that upon unfolding in the presence of Ca^2+^-CaM Trp residues move into a more hydrophobic environment compared to their position in apo-NudT9H. It should be noted that the only reporter tryptophanes are located in NudT9H as CaM does not contain Trp residues.

In order to investigate whether the proposed anchor residue W1355 is the Trp residue that changes into a hydrophobic environment upon binding to Ca^2+^-CaM, we measured tryptophan fluorescence spectra of VL24 peptide (containing W1355 as single reporter) on its own and in complex with excess Ca^2+^-CaM (not containing any Trp residue) (Fig. 2E). The VL24 peptide on its own shows a fluorescence maximum at approx. 355 nm, typical for a solvent exposed tryptophan. In the presence of excess Ca^2+^-CaM a significant blue shift to a maximum at approx. 328 nm occurs, indicating that the Trp residue in the VL24 peptide (corresponding to W1355 in NudT9H) is located in a hydrophobic environment in the complex with Ca^2+^-CaM (Fig. 2E).

Having established that Ca^2+^-CaM seems to bind to partially unfolded or thermally destabilized NudT9H, we next investigated the thermal aggregation profile by dynamic light scattering (DLS) (Fig. 2F). NudT9H in the absence and presence of Ca^2+^-CaM was heated up and the hydrodynamic radius of the particles in solution was monitored as a function of temperature. The transition midpoint for the aggregation reaction increased from 42 °C for NudT9H to 47 °C for NudT9H + Ca^2+^-CaM, indication that Ca^2+^-CaM not only binds to NudT9H during thermal destabilization/unfolding but also stabilizes it against aggregation (“acts as chaperone”) (Fig. 2F).

### 3.3 CaM binding to the NudT9H domain has an effect on the temperature-dependence of the ADPR induced current and activation of TRPM2 by 2′-deoxy-ADPR

**Fig. 3:**
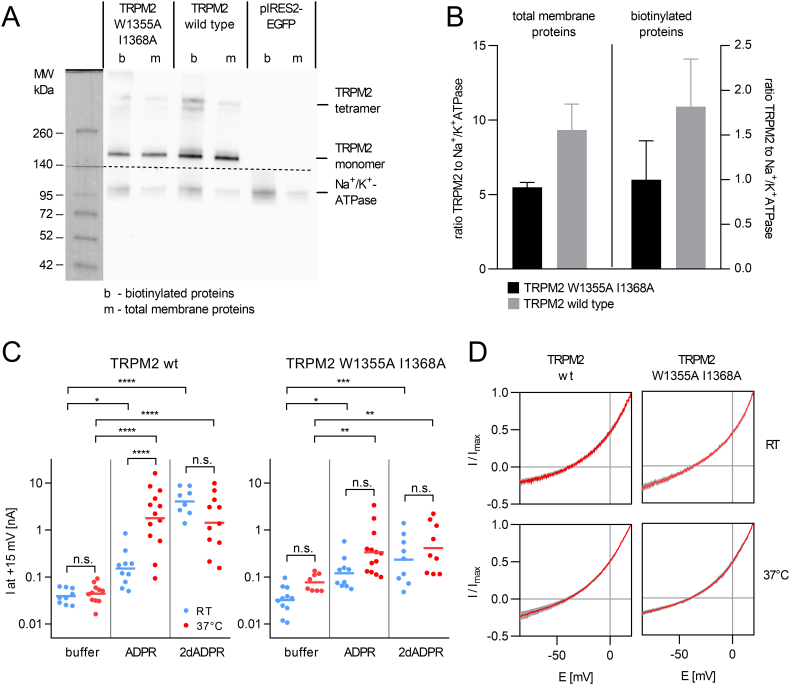
CaM binding to the NudT9H domain has a temperature-dependent effect on the activation of full-length TRPM2 by ADPR and 2′-deoxy-ADPR in HEK293 cells. (A) Mutation of the CaM binding site does not prevent translocation of TRPM2 to the plasma membrane. HEK293 cells were transiently transfected with pIRES2-EGFP (control), pIRES2-EGFP-TRPM2 or pIRES2-EGFP-TRPM2 W1355A/I1368A. After 48 h, proteins of the plasma membrane were biotinylated and isolated from the cells. Afterwards biotinylated proteins were isolated using neutravidine agarose beads. Proteins were separated on a 4–15% SDS–PAGE (10 μg of total membrane proteins and the biotinylated proteins precipitated from 300 μg of membrane proteins) and transferred to a PVDF membrane. The membrane was cut where indicated (dashed line) to probe the lower part for Na^+^/K^+^-ATPase and the upper part for TRPM2. The membrane is from one of three independent experiments. (B) Quantification of the relative expression of wild-type TRPM2 and TRPM2 W1355A/I1368A shows that the expression level of the mutant is 54% of the wild-type. Density of the bands from monomeric TRPM2 were determined using Fiji/ImageJ and were normalized to the density of the Na^+^/K^+^-ATPase bands. The ratio from three independent experiments is indicated as mean ± SEM. (C) Mutation of the CaM binding site in TRPM2 affects the increase in ADPR induced current in response to temperature and activation by 2′-deoxy-ADPR. Points indicate maximum whole cell current at +15 mV from individual cells at RT (blue) or 37 °C (red) (8–13 cells per condition). The pipette solution contained either no nucleotide (buffer) or 100 μM of the indicated agonist. The bar indicates the mean of the log-transformed data. Log transformed data were tested for significant differences using one-way ANOVA and Bonferroni correction (*p < 0.05, **p < 0.01, ***p < 0.001, ****p < 0.0001). (D) The W1355A I1368A mutation does not affect the IV curve of TRPM2 activated by ADPR at room temperature or 37 °C. IV curves were obtained from voltage ramps (from −85 mV to +20 mV) during maximum channel activity and were normalized to the maximum current. Due to the substitution of Na^+^ by NMDG in the bath solution the IV curve shows a reversal potential at roughly −40 mV and a larger outward current. Data are represented as mean with the shaded area indicating the SEM (n = 6–13).

With these results, initially identified using a peptide and then transferred to the NudT9H domain, we next aimed at investigating the effects of CaM binding to NudT9H in the context of full-length TRPM2 and in combination with the agonists ADPR and 2′-deoxy-ADPR. We transiently transfected HEK293 cells with expression vectors for either wild-type TRPM2 or the double mutant TRPM2 W1355A I1368A, in which both anchor residues involved in CaM-binding were mutated to Ala. The channel variant was clearly present in the plasma membrane as judged by a surface biotinylation assay (Fig. 3A), densitometric analysis indicated that the relative expression level of the mutant channel at the plasma membrane was 54% of that of the wild-type channel (Fig. 3B). The fact that a similar ratio between wild-type TRPM2 and TRPM2 W1355A I1368A is observed for total membrane protein and surface biotinylated proteins indicates that the reason is probably not a retention of the mutant channel in intracellular membranes but a generally lower protein expression of the channel variant. We activated TRPM2 with saturating concentrations of either ADPR or 2′-deoxy-ADPR (100 μM) in the pipette solution. In addition we changed the temperature of the bath solution from room temperature to 37 °C.

In the absence of nucleotide agonist there was no significant difference in current between room temperature and 37 °C for cells expressing either wild-type TRPM2 or the double mutant TRPM2 W1355A I1368A. Addition of 100 μM ADPR to the pipette solution resulted in a similar current for wild-type TRPM2 and TRPM2 W1355A I1368A. But while the current of wild-type TRPM2 with 100 μM ADPR in the pipette significantly increased >10-fold (from 150 pA to 2.2 nA, median values) when the temperature was raised from room temperature to 37 °C, there was no significant increase for TRPM2 W1355A I1368A (Fig. 3C). The IV curve, which showed a reversal potential of about -40 mV and a larger outward component due to the replacement of Na^+^ in the bath solution by NMDG was not affected by the W1355A I1368A mutation (Fig. 3D).

For wild-type TRPM2 the current with 100 μM of 2′-deoxy-ADPR in the pipette solution was already significantly higher at room temperature (4.5 nA compared to 150 pA for ADPR, median values) (Fig. 3C) in agreement with our previous results [22]. In contrast to what we observed for ADPR, the current elicited by 2′-deoxy-ADPR did not significantly increase when the temperature was raised from room temperature to 37 °C (4.5 nA compared to 1.4 nA, median values) indicating that the already high open probability induced by a saturating concentration of 2′-deoxy-ADPR does not increase any further when temperature is raised (Fig. 3C). Interestingly and rather unexpectedly, the double mutant did not only affect the temperature dependent increase in current in response to ADPR but also the current induced by 2′-deoxy-ADPR. At both room temperature and 37 °C the current with 2′-deoxy-ADPR in the pipette solution did not significantly differ from that with ADPR (Fig. 3C), indicating that the binding of Ca^2+^-CaM to the CaM binding site in the NudT9H domain is not only required for the heat-induced increase in current in response to ADPR but also for the response of the channel to the superagonist 2′-deoxy-ADPR.

**Supplementary Fig. 1:**
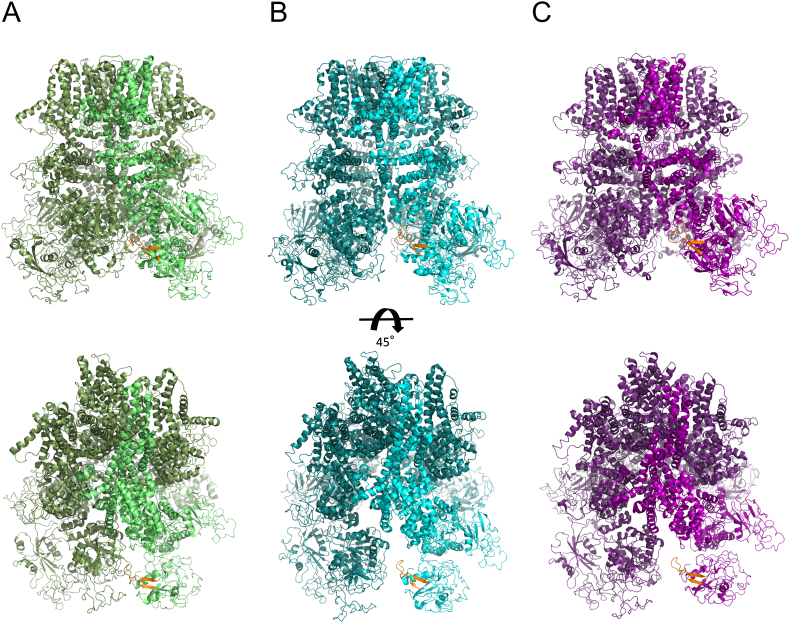
Localization of temperature-dependent CaM-binding site (VL24) in the context of human full-length TRPM2. Structures of hsTRPM2 in different states are displayed in cartoon representation with the CaM-binding region (P-loop in the NudT9H domain) investigated in this study coloured in orange: (A) closed apo state (pdb: 6MIX) in green; (B) primed, ADPR-bound state (pdb: 6MIZ) in cyan; (C) “open”, ADPR- and Ca^2+^-bound state (pdb: 6MJ2) in violet. One monomer in each structure is coloured slightly differently for better visualization and bottom panels are rotated by 45°.

In conclusion, we identified and characterized a novel CaM-binding site in the TRPM2 NudT9H domain and showed that this site could play a role in the temperature-sensitive activation of TRPM2. The cryo-EM structures of hsTRPM2 [27] indicate that the P-loop region in NudT9H, which largely overlaps with the CaM-binding site identified in this manuscript is involved in trans interactions between NudT9H and MHR1/2 of a neighboring subunit in the absence of ADPR and thus most likely not accessible for CaM-binding. In the ADPR-activated state these interactions are broken rendering the CaM-binding site accessible (Suppl. Fig. 1). Interestingly, drTRPM2, which does not respond to heat [44] contains a P-loop deletion and does not show any intersubunit interactions in the cryo-EM structures in either apo or activated states [26]. It is tempting to speculate that higher temperatures might disrupt the interaction between P-loop and MHR1/2 and simultaneously prime the NudT9H domain for CaM-binding. For a more detailed understanding structural studies of TRPM2 in CaM-bound states are required.

The following are the supplementary data related to this article.

## Abbreviations

ADPR: adenosine diphosphate (ADP)-ribose
2dADPR: 2′-deoxy- adenosine diphosphate (ADP)-ribose
BSA: bovine serum albumin
CaM: calmodulin
CaMBD: calmodulin-binding domain
CD: circular dichroism
DLS: dynamic light scattering
drTRPM2: *Danio rerio* TRPM2
DSF: differential scanning calorimetry
GST: Gluthathione S-transferase
hsTRPM2: *Homo sapiens* TRPM2
IMAC: immobilized metal affinity chromatography
IMP: integral membrane protein
ITC: isothermal titration calorimetry
NMDG: *N*‑methyl‑d‑glucamine
NMR: nuclear magnetic resonance
SEC: size-exclusion chromatography
TEV: tobacco etch virus
TMD: transmembrane domain
TROSY: transverse relaxation-optimized spectroscopy
TRPM2: transient receptor potential cation channel, subfamily M, member 2
VL24: peptide with sequence VTHWRRNEDGAICRKSIKKMLEVL containing NudT9H CaM-binding site

## Transparency document

Transparency document Image 1

## References

[1] Faouzi M., Penner R., Nilius B., Flockerzi V. (2014). TRPM2. Handb. Exp. Pharmacol.

[2] Knowles H., Li Y., Perraud A.L. (2013). The TRPM2 ion channel, an oxidative stress and metabolic sensor regulating innate immunity and inflammation. Immunol. Res..

[3] Hara Y., Wakamori M., Ishii M., Maeno E., Nishida M., Yoshida T., Yamada H., Shimizu S., Mori E., Kudoh J., Shimizu N., Kurose H., Okada Y., Imoto K., Mori Y. (2002). LTRPC2 Ca2+-permeable channel activated by changes in redox status confers susceptibility to cell death. Mol. Cell.

[4] Zhang W., Hirschler-Laszkiewicz I., Tong Q., Conrad K., Sun S.-C., Penn L., Barber D.L., Stahl R., Carey D.J., Cheung J.Y., Miller B.A. (2006). TRPM2 is an ion channel that modulates hematopoietic cell death through activation of caspases and PARP cleavage. Am. J. Phys. Cell Physiol..

[5] Partida-Sanchez S., Gasser A., Fliegert R., Siebrands C.C., Dammermann W., Shi G., Mousseau B.J., Sumoza-Toledo A., Bhagat H., Walseth T.F., Guse A.H., Lund F.E. (2007). Chemotaxis of mouse bone marrow neutrophils and dendritic cells is controlled by adp-ribose, the major product generated by the CD38 enzyme reaction. J. Immunol..

[6] Yamamoto S., Shimizu S., Kiyonaka S., Takahashi N., Wajima T., Hara Y., Negoro T., Hiroi T., Kiuchi Y., Okada T., Kaneko S., Lange I., Fleig A., Penner R., Nishi M., Takeshima H., Mori Y. (2008). TRPM2-mediated Ca^2+^ influx induces chemokine production in monocytes that aggravates inflammatory neutrophil infiltration. Nat. Med..

[7] Wehrhahn J., Kraft R., Harteneck C., Hauschildt S. (2010). Transient receptor potential melastatin 2 is required for lipopolysaccharide-induced cytokine production in human monocytes. J. Immunol..

[8] Melzer N., Hicking G., Göbel K., Wiendl H. (2012). TRPM2 cation channels modulate T cell effector functions and contribute to autoimmune CNS inflammation. PLoS One.

[9] Di A., Gao X.-P., Qian F., Kawamura T., Han J., Hecquet C., Ye R.D., Vogel S.M., Malik A.B. (2011). The redox-sensitive cation channel TRPM2 modulates phagocyte ROS production and inflammation. Nat. Immunol..

[10] Sawamura S., Shirakawa H., Nakagawa T., Mori Y., Kaneko S., Emir T.L.R. (2017). TRP channels in the brain: what are they there for?. Neurobiol.

[11] Sita G., Hrelia P., Graziosi A., Ravegnini G., Morroni F. (2018). TRPM2 in the brain: role in health and disease. Cell.

[12] Song K., Wang H., Kamm G.B., Pohle J., Reis F. de C., Heppenstall P., Wende H., Siemens J. (2016). The TRPM2 channel is a hypothalamic heat sensor that limits fever and can drive hypothermia. Science.

[13] Tan C.-H., McNaughton P.A. (2016). The TRPM2 ion channel is required for sensitivity to warmth. Nature.

[14] McHugh D., Flemming R., Xu S.Z., Perraud A.L., Beech D.J. (2003). Critical intracellular Ca^2+^ dependence of transient receptor potential melastatin 2 (TRPM2) cation channel activation. J. Biol. Chem..

[15] Starkus J., Beck A., Fleig A., Penner R. (2007). Regulation of TRPM2 by extra- and intracellular calcium. J. Gen. Physiol..

[16] Csanády L., Törocsik B. (2009). Four Ca^2+^ ions activate TRPM2 channels by binding in deep crevices near the pore but intracellularly of the gate. J. Gen. Physiol..

[17] Togashi K., Hara Y., Tominaga T., Higashi T., Konishi Y., Mori Y., Tominaga M. (2006). TRPM2 activation by cyclic ADP-ribose at body temperature is involved in insulin secretion. EMBO J..

[18] Zhang W., Tong Q., Conrad K., Wozney J., Cheung J.Y., Miller B.A. (2007). Regulation of TRP channel TRPM2 by the tyrosine phosphatase PTPL1. Am. J. Phys. Cell Physiol..

[19] Starkus J.G., Fleig A., Penner R. (2010). The calcium-permeable non-selective cation channel TRPM2 is modulated by cellular acidification. J. Physiol..

[20] Perraud A.L., Fleig A., Dunn C.A., Bagley L.A., Launay P., Schmitz C., Stokes A.J., Zhu Q., Bessman M.J., Penner R., Kinet J.P., Scharenberg A.M. (2001). ADP-ribose gating of the calcium-permeable LTRPC2 channel revealed by Nudix motif homology. Nature.

[21] Tóth B., Iordanov I., Csanády L. (2015). Ruling out pyridine dinucleotides as true TRPM2 channel activators reveals novel direct agonist ADP-ribose-2′-phosphate. J. Gen. Physiol..

[22] Fliegert R., Bauche A., Wolf Pérez A.-M., Watt J.M., Rozewitz M.D., Winzer R., Janus M., Gu F., Rosche A., Harneit A., Flato M., Moreau C., Kirchberger T., Wolters V., Potter B.V.L., Guse A.H. (2017). 2′‑Deoxyadenosine 5′‑diphosphoribose is an endogenous TRPM2 superagonist. Nat. Chem. Biol..

[23] Tong Q., Zhang W., Conrad K., Mostoller K., Cheung J.Y., Peterson B.Z., Miller B.A. (2006). Regulation of the transient receptor potential channel TRPM2 by the Ca^2+^ sensor calmodulin. J. Biol. Chem..

[24] Zhang Z., Tóth B., Szollosi A., Chen J., Csanády L. (2018). Structure of a TRPM2 channel in complex with Ca^2+^ explains unique gating regulation. elife.

[25] Luo Y., Yu X., Ma C., Luo J., Yang W. (2018). Identification of a novel EF-loop in the N-terminus of TRPM2 channel involved in calcium sensitivity. Front. Pharmacol..

[26] Huang Y., Winkler P.A., Sun W., Lü W., Du J. (2018). Architecture of the TRPM2 channel and its activation mechanism by ADP-ribose and calcium. Nature.

[27] Wang L., Fu T.-M., Zhou Y., Xia S., Greka A., Wu H. (2018). Structures and gating mechanism of human TRPM2. Science.

[28] Kashio M., Tominaga M. (2017). The TRPM2 channel: a thermo-sensitive metabolic sensor. Channels.

[29] Veith K., Martinez Molledo M., Almeida Hernandez Y., Josts I., Nitsche J., Löw C., Tidow H. (2017). Lipid-like peptides can stabilize integral membrane proteins for biophysical and structural studies. Chembiochem.

[30] Lescop E., Schanda P., Brutscher B. (2007). A set of BEST triple-resonance experiments for time-optimized protein resonance assignment. J. Magn. Reson..

[31] Johnson B.A. (2004). Using NMRView to visualize and analyze the NMR spectra of macromolecules. Methods Mol. Biol..

[32] Kirchberger T., Moreau C., Wagner G.K., Fliegert R., Siebrands C.C., Nebel M., Schmid F., Harneit A., Odoardi F., Flügel A., Potter B.V.L., Guse A.H. (2009). 8-Bromo-cyclic inosine diphosphoribose: towards a selective cyclic ADP-ribose agonist. Biochem. J..

[33] Schindelin J., Arganda-Carreras I., Frise E., Kaynig V., Longair M., Pietzsch T., Preibisch S., Rueden C., Saalfeld S., Schmid B., Tinevez J.-Y., White D.J., Hartenstein V., Eliceiri K., Tomancak P., Cardona A. (2012). Fiji: an open-source platform for biological-image analysis. Nat. Methods.

[34] Minhas F. ul A.A., Ben-Hur A. (2012). Multiple instance learning of calmodulin binding sites. Bioinformatics.

[35] Hoeflich K.P., Ikura M. (2002). Calmodulin in action: diversity in target recognition and activation mechanisms. Cell.

[36] Tidow H., Nissen P. (2013). Structural diversity of calmodulin binding to its target sites. FEBS J..

[37] Yap K.L., Kim J., Truong K., Sherman M., Yuan T., Ikura M. (2000). Calmodulin target database. J. Struct. Funct. Genom..

[38] Babu Y.S., Bugg C.E., Cook W.J. (1988). Structure of calmodulin refined at 2.2 Å resolution. J. Mol. Biol..

[39] Ikura M., Clore G.M., Gronenborn A.M., Zhu G., Klee C.B., Bax A. (1992). Solution structure of a calmodulin-target peptide complex by multidimensional NMR. Science.

[40] Meador W.E., Means A.R., Quiocho F.A. (1992). Target enzyme recognition by calmodulin: 2.4 a structure of a calmodulin-peptide complex. Science.

[41] Meador W.E., Means A.R., Quiocho F.A. (1993). Modulation of calmodulin plasticity in molecular recognition on the basis of x-ray structures. Science.

[42] Tidow H., Poulsen L.R., Andreeva A., Knudsen M., Hein K.L., Wiuf C., Palmgren M.G., Nissen P. (2012). A bimodular mechanism of calcium control in eukaryotes. Nature.

[43] Shen B.W., Perraud A.L., Scharenberg A., Stoddard B.L. (2003). The crystal structure and mutational analysis of human NUDT9. J. Mol. Biol..

[44] Nam Tran H., Hederih J., Numata T., Mori M.X., Maegawa S., Hosokawa H., Mori Y. (2018). Functional charaterization of zebrafish transient receptor potential melastatin 2. Biophys. J..

